# Prognostic factors and multidisciplinary treatment modalities for brain metastases from colorectal cancer: analysis of 93 patients

**DOI:** 10.1186/s12885-015-1933-2

**Published:** 2015-11-16

**Authors:** Xiao-Dong Gu, Yan-Tao Cai, Yi-Ming Zhou, Zhen-Yang Li, Jian-Bin Xiang, Zong-You Chen

**Affiliations:** 1 Department of General Surgery, Huashan Hospital, Fudan University, Shanghai, 200040 China; 2 Department of General Surgery, Shanghai Ninth People’s Hospital, Shanghai JiaoTong University School of Medicine, Shanghai, 200011 China

**Keywords:** Colorectal cancer, Brain metastases, Surgery, Radiotherapy, Prognosis

## Abstract

**Background:**

The purpose of this study was to review patient characteristics and evaluate the potential factors affecting prognosis in cases of brain metastasis (BM) from colorectal cancer (CRC).

**Methods:**

We retrospectively reviewed 93 cases of BM from CRC in our hospital. Patient demographics, neurologic symptoms, and location and number of BMs were recorded. Factors analyzed included: age; sex; Karnofsky performance score; number of BMs; presence of extracranial metastases; dimensions; location of tumors; treatment modalities.

**Results:**

The overall 1- and 2-year survival rates were 27.7 and 9.9 %. On multivariate analysis, the number of BMs, extracranial metastases and the initial treatment modalities were found to be independent prognostic factors for overall survival. Patients treated with surgical resection followed by WBRT or SRS had an improved prognosis relative to those treated with surgery alone (*P* = 0.02 and *P* = 0.02, respectively). No significance difference in survival rate was found between patients treated with SRS alone or SRS plus WBRT (*P* = 0.11).

**Conclusions:**

Surgical resection of BMs from CRC in selected patients may help prolong survival. Additional radiotherapy following surgery is valuable in improving prognosis. Extracranial metastasis, multiple BM lesions and initial non operation can be considered as independent factors associated with poor prognosis.

## Background

Colorectal cancer (CRC) is the second-leading cause of cancer-related deaths after lung cancer [1]. Morbidity associated with CRC continues to increase as a result of the expanding use of CRC screening, improved diagnostic techniques, and the development of multidisciplinary management [2]. Approximately 50 % of CRC patients will die of metastatic disease [3]. In CRC cases, the incidence of brain metastasis (BM) is much lower than at other common metastatic sites such as the lung, liver, and peritoneal cavity; BM accounts for only 2–3 % of cases with pathological diagnosis at autopsy or using surgical resection [4]. As a result of longer survival times owing to the improved therapeutic response of the primary tumor, the incidence of BMs may be expected to increase. Defined as a terminal-stage phenomenon, the prognosis of BM remains pessimistic with a median survival time of 1–4 months [5, 6]. Alternative approaches in treating BM such as surgical resection of the metastatic site, stereotactic radiosurgery (SRS), and whole brain radiation therapy (WBRT) have been used.

The present study was conducted to analyze the clinical characteristics and outcomes of treatment modalities in patients with BM from CRC, to identify independent prognostic factors, and to provide information on related clinical experience of treatment.

## Methods

### Patient characteristics

Between 2001 and 2011, patients diagnosed with BM from CRC at the Huashan Hospital (Shanghai, China) were included in this study. Data were obtained using the medical records system. Patients included in the study had to have undergone radical resection of the primary tumor. Pathological reports regarding primary CRC were confirmed. BM was diagnosed by means of autopsy reports and surgical pathology, or by clinical diagnosis. Relevant information was reviewed with respect to the following factors: i) patient demographics (age, sex, date of diagnosis, Karnofsky performance score [KPS], location of primary tumor, and stage of primary tumor); ii) BM characteristics (date of BM diagnosis, BM location, number of BM sites, primary neurologic symptoms, and type of treatment for BM); and iii) extracranial metastasis site and number of treatments. Patients were followed up at outpatient clinics, in addition to phone calls, mail, and e-mails. Written informed consent was obtained from all patients or their guardians. This study was approved by the ethics committee of Huashan Hospital of Fudan University.

### Treatment

Treatment modalities included surgical resection, SRS, and WBRT. The treatment program was designed according to the number and location of BMs, the presence of extracranial metastases, and the patient’s general condition.

### Statistical analysis

Statistical analysis was performed using the SPSS 19.0 statistical software package (SPSS Inc., Chicago, IL, USA). The Chi-square test was used to analyze group comparisons where appropriate. Overall survival (OS) was set from the day of BM diagnosis to the termination of the records (day of death or last follow-up date). OS was analyzed using the Kaplan–Meier method and evaluated by means of the log-rank test. Assessment of the individual factors impacting on survival was accomplished using the log-rank test with a significance level of *P* = 0.05. The Cox proportional-hazards model was used to evaluate the impact of multiple factors selected from individual ones.

## Results

### Patient characteristics

Ninety-three patients were identified in this study, including 57 men and 36 women. The median patient age at diagnosis of BM from CRC was 56 years. The primary location of CRC was the colon in 44 patients and the rectum in 49 (Table 1). The characteristics of BM are summarized in Table 2, including the primary neurologic symptoms, KPS, number of BMs, and location of BM. A total of 37 (39.8 %) patients had a solitary BM lesion while the other 56 (60.2 %) had multiple lesions. Extracranial metastasis was detected in 57 (61.3 %) patients, including lung in 57 (61.3 %), liver in 17 (18.3 %), and bone in 12 (12.9 %). Lung metastasis was observed in every patient apart from those who only had BM in this study. Twenty-nine patients had multiple extracranial metastatic sites (17 with lung plus liver and 12 with lung plus bone) (Table 3).

**Table 1:** Patient demographics

Characteristic	Patients, n (%)
Gender	
Male	57, (61.3 %)
Female	36, (38.7 %)
Age	
<60	50, (53.8 %)
≥60	43, (46.2 %)
Primary tumor site	
Ascending colon	11, (11.8 %)
Transverse colon	3, (3.2 %)
Descending colon	7, (7.5 %)
Sigmoid colon	23, (24.7 %)
Rectum	49, (52.8 %)

**Table 2:** Characteristics of BM from CRC

Characteristic	Patients, n (%)
Primary neurologic symptom of BM	
Symptom of ICP (headache, nausea)^a^	43, (46.2 %)
Difficulties of balance, gait or speech	48, (51.6 %)
Asymptomatic	2, (2.2 %)
KPS	
>70	33, (35.5 %)
60 ~ 70	42, (45.2 %)
<60	18, (19.3 %)
Number of BM	
1	37, (39.8 %)
2	6, (6.5 %)
3	2, (2.1 %)
≥4	48, (51.6 %)
Location of solitary BM	
Frontal lobe	12, (32.4 %)
Temporosphenoid lobe	4, (10.8 %)
Occipital lobe	2, (5.4 %)
Parietal lobe	3, (8.1 %)
Cerebellum	15, (40.6 %)
Others	1, (2.7 %)
Location of multiple BM	
Supratentorial	25, (44.7 %)
Infratentorial	11, (19.6 %)
Supratentorial + Infratentorial	20, (35.7 %)

^a^*ICP* increased intracranial pressure

**Table 3:** Extracranial metastatic burden

Characteristic	Patients, n (%)
Extracranial metastatic site	
Absent	36, (38.7 %)
Lung	57, (61.3 %)
Liver	17, (18.3 %)
Bone	12, (12.9 %)
Metastatic burden	
BM/absent	36, (38.7 %)
BM/lung	28, (30.1 %)
BM/lung/liver	17, (18.3 %)
BM/lung/bone	12, (12.9 %)

### Therapy

The treatments received by patients were as follows: surgery only, 25 (26.9 %); SRS only, 9 (9.7 %); WBRT only, 22 (23.7 %); surgery plus WBRT, 19 (20.4 %); surgery plus SRS, 11 (11.8 %); and SRS plus WBRT, 7 (7.5 %). Twenty-seven patients underwent SRS during their treatment course, nine underwent SRS as their primary treatment for BM, and 18 underwent SRS as salvage therapy. The prescription dose ranged from 1200 centigray (cGy) to 2400 cGy. Forty-eight patients received WBRT during their management; in 22 of these patients WBRT was the primary treatment and in the other 26 WBRT was used as salvage therapy. Patients generally received 3000 cGy in ten fractions (patients with a KPS < 70) or 4000 cGy in 20 fractions (patients with a KPS ≥70).

### Outcomes and prognostic factors

**Fig. 1:**
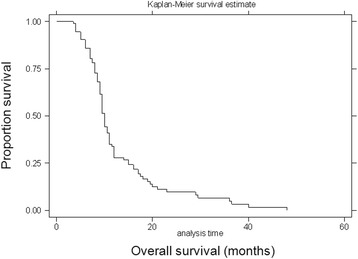
Survival curve for patients diagnosed as BM from CRC. Overall survival in the 93 patients after diagnosis of BM from CRC. The overall 1- and 2-year survival rates were 27.7 and 9.9 %, respectively

The median survival time after the diagnosis of BM from CRC for all 93 patients was 9.6 months. According to the follow-up data, the overall 1- and 2-year survival rates were 27.7 and 9.9 %, respectively (Fig. 1). Using the log-rank test, the following factors were found to be significant univariate predictors of survival: number of BMs (*P* < 0.01), presence of extracranial metastasis (*P* < 0.01), and the initial treatment modalities (*P* < 0.01) (Table 4). When evaluated using multivariate analysis, the number of BMs, presence of extracranial metastasis and the initial treatment modalities were proved to be independent prognostic factors regarding the overall survival of patients with BM from CRC (Table 5).

**Table 4:** Univariate predictors of survival in patients with BM from CRC

Variables	Number	Median (months)	*χ*2	*P*
Age				
<60	50	14	3.626	0.06
≥60	43	9		
Gender				
Male	57	9	1.124	0.29
Female	36	11		
KPS				
≥70	33	13	2.168	0.14
<70	60	9		
Number of BM				
Solitary	37	10	11.395	<0.01
Multiple	56	6		
Greatest tumor dimension, cm				
>3 cm	58	10	0.117	0.73
≤3 cm	35	9		
Location of BM				
Supratentorial	46	15	4.685	0.20
Infratentorial	26	11		
Supratentorial + Infratentorial	20	12		
Others	1	9		
Extracranial metastasis				
Yes	57	7	19.211	<0.01
No	36	13		
First treatment				
Surgery	55	15	38.206	<0.01
SRS	22	10		
WBRT	16	6

**Table 5:** Multivariate predictors of survival of BM (screened from univariate predictors with *P* < 0.05)

	Multivariate analysis		
Variables	Relative risk	95 % CI^a^	*P*
Number of BM	0.489	−3.43 ~ 4.56	0.01
Extracranial metastasis	1.418	0.77-2.52	0.03
First treatment	5.471	1.48 ~ 9.45	<0.01

^a^*CI* confidence interval

**Fig. 2:**
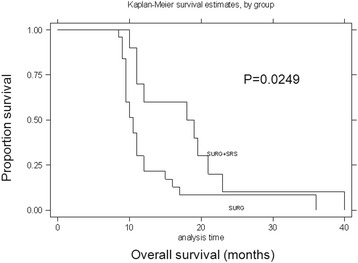
Survival curves for surgery alone versus surgery plus SRS treatment modalities for BM. Kaplan–Meier survival curves for patients stratified by treatment modality for BM (surgery alone versus surgery plus SRS). There was a significant correlation between surgery alone and surgery plus SRS (*P* = 0.02, log-rank test)

**Fig. 3:**
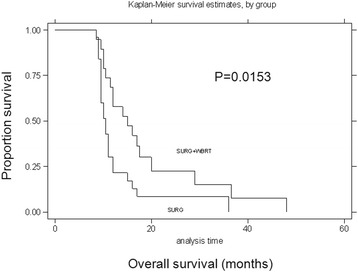
Survival curves for surgery alone versus surgery plus WBRT treatment modalities for BM. Kaplan–Meier survival curves for patients stratified by treatment modality for BM (surgery alone versus surgery plus WBRT). There was a significant correlation between surgery alone and surgery plus WBRT (*P* = 0.02, log-rank test)

**Fig. 4:**
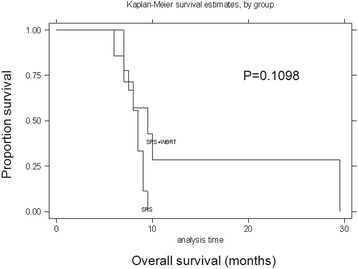
Survival curves for SRS alone versus SRS plus WBRT treatment modalities for BM. Kaplan–Meier survival curves for patients stratified by treatment modality for BM (SRS alone versus SRS plus WBRT). There was no significant correlation between SRS alone and SRS plus WBRT (*P* = 0.11, log-rank test)

Significant differences concerning patient prognosis were evident between the treatment modalities (Figs. 2, 3 and 4). Surgery combined with adjuvant radiotherapy achieved higher OS times than surgery alone (surgery vs surgery plus SRS, *P* = 0.02; surgery vs surgery plus WBRT, *P* = 0.02). For patients who received SRS, additional WBRT did not achieve a therapeutic advantage (SRS vs SRS plus WBRT, *P* = 0.11).

## Discussion

BM from primary tumors in the colon and rectum is relatively rare as compared with BM from other sites such as the lung and breast [7]. CRC is the most common primary lesion among BMs originating from tumors in the gastrointestinal tract; indeed, a recent systematic review found that 79.9 % (2028/2538) of BMs originated from tumors in the gastrointestinal tract [8]. BM occurs among approximately 10 % of CRC patients during a subsequent course of CRC, and in 2–3 % of patients BM was the first clinical manifestation [9, 10]. Viewed as a terminal stage of cancer, BM from CRC is associated with poor prognosis with an OS time of <6 months [11, 12]. Development in surgical technique and chemoradiotherapy has improved the control of primary CRC, and has extended OS time. These improvements have increased the chances of CRC patients who had previously died from extracranial systematic disease of developing BM. In addition, growth in the use of imaging techniques has resulted in increased detection of previously occult BM lesions. Although tumor recurrence and extracranial metastases remain the leading cause of death in CRC patients, BM has a growing status due to better prognosis of CRC patients.

Lung metastases in CRC are less common than liver metastases in CRC. However, in association with BM, lung metastases are reportedly more commonly seen. This concurred with the findings in our series. In previous studies, the incidence of combined lung metastases has been reported to range from 55 to 85 % [13, 14]; it was 61.3 % in the present study. Cascino and colleagues specifically listed the following three pathways as potential routes of BM through circulation [4]: (1) From the rectal venous plexus to the inferior vena cava, (2) Through the Batson spinal venous plexus, (3) Through the portal vein, liver, and lung. The risk of subsequent metastasis to the brain increases once CRC cells metastasize to the lung and liver. This suggests that metastasis through the portal vein and subsequently through the liver and lungs could be the major route for BM from CRC [9]. The mechanism associated with the development of BM remains controversial with several molecular pathways and markers under suspicion [2, 15–17].

In the current study, 72 (77.4 %) cases of BM were found to have originated from the sigmoid colon or rectum. A similar distribution was also observed in several studies from different centers, with approximately 60 % of BM occurring in CRC cases [18, 19]. Some researchers have hypothesized that tumors located in the rectal site have a higher incidence of lung metastases because of differences in vascular anatomy [20]. Hammoud et al. [21] analyzed 150 patients with BM from CRC and found that proximal colonic lesions were more often associated with liver metastasis, which resulted in shorter survival times as compared with distal lesions. This finding suggested that most CRC patients with proximal colonic lesions die from extracranial metastasis before BM develops. Patients with distal colon cancer have a greater chance of developing BM before cancer-related death [11].

In the present study, the number of BM lesions was revealed to be an independent factor affecting patient prognosis, which was similar to the finding of previous studies [6, 22]. Several studies have reported that surgical resection is the most efficacious and expeditious treatment for BM from CRC, and improvement in prognosis has been found [18, 23]. Patients with multiple sites of BM have a lower chance of undergoing brain surgery, in consideration of the surgical difficulty and potential complications. In addition, poor systemic status is more common in this group of patients, which may affect the more palliative treatment strategies. In our study, radiotherapy, WBRT, or SRS, followed by surgical resection led to significantly improved survival relative to surgery alone. This study has found significant improved survival with surgical resection. This may reflect a selection bias, as patients with limited systemic metastatic load, solitary brain lesions in surgically accessible areas, and/or a life expectancy of 6 months or greater are more likely to be selected for curative resection. On the other hand, it highlights the possibility that brain metastases, if treated aggressively, can lead to improved survival.

WBRT or SRS alone are considered as palliative treatments for relieving the neurological symptoms of patients who are unfit for surgery. In the present study, selection bias was obviously a matter of prime importance. Patients with multiple BM lesions were more suitable for receiving WBRT than SRS. Patients with increased numbers of BM lesions have been proved to have shorter survival times, and this may explain the difference in the outcomes of the radiotherapies. SRS followed by additional WBRT exhibited no advantage over SRS alone in the current study; this was also noted by Schoeggl et al. [24]. Whether the treatment option will provide benefit over other treatment strategies, should be verified in prospective studies.

## Conclusions

BM remains a terminal-stage phenomenon of CRC. The findings of the present study indicated that surgical resection of BM in selected patients may prolong survival. Additional radiotherapy following surgery is valuable in providing additional improvement in prognosis. Combined extracranial metastasis, more than three BM lesions and the initial no operation are considered independent factors associated with poor prognosis.

## Abbreviations

CRC: colorectal cancer
BM: brain metastasis
SRS: stereotactic radiosurgery
WBRT: whole brain radiation therapy
OS: overall survival
cGy: centigray
KPS: karnofsky performance score
